# Clinical characteristics of catheter-related infection in patients with chronic renal failure End Stage Renal failure undergoing semi-permanent catheter placement during maintenance hemodialysis through tunnelled cuffed hemodialysis catheter

**DOI:** 10.12669/pjms.38.6.4834

**Published:** 2022

**Authors:** Jun Dou, Xuebing Wu, Hua Ao, Qiuling Zhang, Ming Li

**Affiliations:** 1 Jun Dou, Department of Nephrology, The Third Clinical Medical College of Three Gorges University, Sinopharm Gezhouba Central Hospital, Yichang 443002, Hubei, China; 2 Xuebing Wu, Department of Nephrology, The Third Clinical Medical College of Three Gorges University, Sinopharm Gezhouba Central Hospital, Yichang 443002, Hubei, China; 3 Hua Ao, Department of Nephrology, The Third Clinical Medical College of Three Gorges University, Sinopharm Gezhouba Central Hospital, Yichang 443002, Hubei, China; 4 Qiuling Zhang, Department of Nephrology, The Third Clinical Medical College of Three Gorges University, Sinopharm Gezhouba Central Hospital, Yichang 443002, Hubei, China; 5 Ming Li, Department of Nephrology, The Third Clinical Medical College of Three Gorges University, Sinopharm Gezhouba Central Hospital, Yichang 443002, Hubei, China

**Keywords:** Chronic renal failure, Hemodialysis, Semi-permanent catheter, Catheter-related infection

## Abstract

**Objective:**

To analysis the relevant infections and risk factors of patients undergoing hemodialysis semi-permanent catheter (tunneled cuffed) placement during for maintenance hemodialysis.

**Methods:**

A total of 158 patients with chronic renal failure (CRF) End stage renal failure (ESRF) treated in our hospital from September 2018 to September 2021 were retrospectively analyzed. All the patients underwent semi-permanent catheter placement during maintenance hemodialysis. The occurrence of catheter-related infections in the patients were recorded. The patients with catheter-related infections were included in the infection group, and the others without infection in the non-infection group. The differences in hypertension, gender, diabetes, age, catheter indwelling time and dialysis time between the two groups were analyzed, and the distribution of pathogens in the patients with infections was analyzed.

**Results:**

The patients were followed up for 13 to 36 months, with an average of (22.18 ± 6.09) months. Among the 158 patients who underwent going semi-permanent catheter placement, 42 (26.58%) presented semi-permanent catheter-related infections, including four cases of catheter-related bacteremia, 16 cases of tunnel infection and 22 cases of catheter exit-site infection. Among total of 42 strains of pathogens were isolated from the 42 patients with catheter-related infections, including 243 strains of Gram-positive cocci were identified in 24/42(57.14%), and 163 strains of Gram-negative bacilli were identified 16/42(38.10%) and one starin of fungus was identified in 2/42 patients. Statistically significant differences were found in dialysis duration time, hypoalbuminemia, average mean age, diabetes and catheter indwelling time between patients with and without catheter-related infections (*P* < 0.05). Hypoalbuminemia, catheter indwelling time and diabetes were risk factors for catheter-related infections (*P* < 0.05).

**Conclusions:**

Patients with ESRF CRF are at risk and prone to catheter-related infections during hemodialysis using catheter, mainly tunnel infection and catheter exit-site infection. Gram-positive cocci are the main pathogens. Hypoalbuminemia, too long catheter indwelling time and diabetes are the risk factors for infections.

## INTRODUCTION

Chronic renal failure (CRF) is one of the common clinical diseases with high morbidity and mortality.^1^ With the development of the disease, it can reduce the glomerular filtration rate and destroy the nephron to a certain extent, and eventually cause irreversible kidney damage, or even renal failure.^2^ Therefore, it is important to implement effective treatment as soon as possible, which has a positive effect on improving the quality of life and prognosis of patients.^3^ A reliable and stable vascular approach is the necessary condition for maintenance hemodialysis in patients with ESRF, but the condition of such vessels is often poor, resulting in impossible arteriovenous fistula surgery.^4^,^5^ Semi-permanent catheter is the first choice for the establishment of vascular approach in such patients with diseased vessles and in elderly patients with CRF, characterized by large volume of dialyzed blood, rapid and simple operation and less pain. However, catheter is an exogenous foreign body and catheterization is an invasive operation, which may lead to catheter-related infections. This is also the main cause of clinical death in patients during maintenance hemodialysis.^6^,^7^ Therefore, in this study, the relevant infection and risk factors of patients undergoing semi-permanent catheter placement during maintenance hemodialysis were analyzed, so as to provide some reference for the clinical diagnosis and treatment.

## METHODS

A total of 158 patients with ESRF CRF treated in our hospital from September 2018 to September 2021 were retrospectively analyzed. For all the patients with failure to achieve AV fistula or AV graft in internal fistula puncture caused by vascular factors and unable re-establishment of internal fistula, semi-permanent catheter was placed for during maintenance hemodialysis. The patients aged from 62 to 78 years (average, 63.29 ± 3.10 years), including 96 males and 62 females, with the dialysis duration time of 24-55 months (average, 38.62 ± 10.63 months). The primary diseases included polycystic kidney disease (n = 4), diabetic nephropathy (n = 60), obstructive nephropathy (n = 2), hypertensive nephropathy (n = 56), lupus nephritis (n = 4) and glomerulonephritis (n = 32).

### Ethical approval

The study was approved by the Institutional Ethics Committee of the Third Clinical Medical College of Three Gorges University, Sinopharm Gezhouba Central Hospital on October 10, 2019 (No. [2019]036), and written informed consent was obtained from all participants.

### Catheter placement

Under local anesthesia, all the patients were catheterized via the right jugular vein and intubated by Seldinger’s technique using the Dual-D-lumen Palindrome H Chronic Catheter Kit (heparin-coated, COVIDIEN, USA), with the length of 35-40 cm and 14.5 Fr. The patients were in the supine position with the head turned to the opposite side. At the puncture point, the artery was pressed and pushed medially with one hand, and the needle was inserted at 30°-45° between the skin and the lateral carotid artery with the other hand. When the outflow was dark red venous blood, the puncture needle was inserted with guide wire fixation. The cutaneous outlet and subcutaneous position of the catheter were evaluated. After the catheter was introduced by a tunnel needle, the catheter was expanded and inserted along the guide wire. The cutaneous outlets of the Cuff and catheter were kept 2-3 cm away, the subcutaneous tunnel was maintained within 10-15 cm, and the tip of the catheter was superficially located at the 3rd-4th rib. The skin was sutured and bandaged with a sterile dressing.

The occurrence of catheter-related infections in the patients were recorded. The patients with catheter-related infections were included in the infection group, and the others without infection in the non-infection group. The differences in hypertension, gender, diabetes, age, catheter indwelling time and dialysis time between the two groups were analyzed. The distribution of pathogens in the patients with infections was analyzed. The strains were isolated and cultured using the BACTEC 9050 culture system (Shanghai Qianchen Biotechnology Co., Ltd.), and the species and number of strains were counted.

### Statistical analysis

The data were analyzed using SPSS 19.0. The qualitative data were expressed as frequencies and percentages. The quantitative data were expressed as mean±standard deviation and analyzed by *t*-test. The factors for catheter-related infections were analyzed with Cox proportional hazards regression. P < 0.05 was considered as significant difference.

## RESULTS

The patients were followed up for 13 to 36 months, with an average of (22.18 ± 6.09) months. Among the 158 patients undergoing semi-permanent catheter placement, 42 (26.58%) presented semi-with permanent catheter-related infections, including four cases of catheter-related bacteremia, 16 cases of tunnel infection and 22 cases of catheter exit-site infection.

Among total of 42 strains of pathogens were isolated from the 42 patients with catheter-related infections, include ing 24 patients were with three strains of Gram-positive cocci (57.14%) and 16 patients were with three strains of Gram-negative bacilli (38.10%), and two patients were with one strain of fungus as shown in Table-I. Statistically significant differences were found in dialysis time, hypoalbuminemia, average age, diabetes and catheter indwelling time between patients with and without catheter-related infections (*P* < 0.05), as seen in Table-II. The factors with differences in the single-factor analysis were included in the Cox proportional hazards regression model, revealing that hypoalbuminemia, catheter indwelling time and diabetes were risk factors for catheter-related infections (*P* < 0.05, Table-III).

**Table I:** Distribution of pathogens in patients with catheter-related infections.

Distribution of pathogenic strains	Number of strains	Proportion (%)
Gram-negative bacilli	16	38.10
Pseudomonas aeruginosa	6	14.29
Escherichia coli	6	14.29
Acinetobacter baumannii	4	9.52
Gram-positive cocci	24	57.14
Enterococcus faecalis	2	4.76
Staphylococcus epidermidis	8	19.05
Staphylococcus aureus	14	33.33
Fungi	2	4.76
Candida paraplanatus	2	4.76

**Table II:** Single-factor analysis of catheter-related infections in patients.

Group	Dialysis time (month)	Male / Female	Hypoalbuminemia	Average age (years)	Hypertension	Diabetes	Catheter indwelling time (month)
Patients with infections (n = 42)	45.18± 6.20	22/20	30 (71.43)	66.75± 2.34	16 (38.10)	36 (85.71)	26.50 ± 5.40
Patients without infections (n = 116)	34.36± 6.31	74/42	60 (51.72)	62.47± 2.09	40 (34.48)	24 (20.69)	16.22 ± 5 .37
t/²	16.387	0.275	9.342	8.551	0.076	15.432	20.419
P	< 0.05	> 0.05	< 0.05	< 0.05	> 0.05	< 0.05	< 0.05

**Table III:** Regression analysis of factors influencing catheter-related infections.

Influencing factor	Regression coefficient	*P*	*OR*	*95%CI*
Hypoalbuminemia	1.261	0.020	0.339	1.258~1.880
Average age	0.668	0.151	1.022	0.668~1.149
Diabetes	1.169	0.002	4.209	2.150~6.020
Catheter indwelling time	1.159	0.005	2.907	1.200~4.370
Dialysis time	0.560	0.217	0.969	0.568~1.238

## DISCUSSION

This study showed that among the 158 patients undergoing semi-permanent catheter placement, 42 (26.58%) presented semi-permanent catheter-related infections, including four cases of catheter-related bacteremia, 16 cases of tunnel infection and 22 cases of catheter exit-site infection, indicating that the main site of bacterial invasion was is the skin where is catheterized, was inserted which is related to catheter-related operation before and after dialysis and the wound at catheter puncture site. Consequently, the aseptic operation should be strictly carried out when sealing catheter with heparin and before and after dialysis, so as to reduce the risk of infections.^8^,^9^ The main pathogens were Gram-positive cocci, which is consistent with the relevant research at home and abroad.^10^,^11^ Relevant studies have shown that the main pathogens of deep venous catheter-related infection in most patients during maintenance hemodialysis are Gram-positive cocci carried in the nasal cavity and skin, mainly Staphylococcus aureus and Staphylococcus epidermidis.^12^-^16^ In addition, with the combination and variety of clinical antibiotics increase, the drug resistance and species of pathogens in patients are also increasing. In this study, there were two cases of fungal infection, suggesting that familiarity with the common flora of patients has a guiding role in the treatment, so that the infections can be controlled as soon as possible.

Patients with diabetes will suffer from hyperglycemia for a long time, leading to decreased transformation rate of lymphocytes, increased blood viscosity, reduced complements, antibodies and immunoglobulins, faster protein decomposition and decreased synthesis, which will affect the body’s defense function and immune response.^17^-^20^ It has been shown that if the body does not control blood glucose well, it will result in a decrease in complement C4 content, affecting the phagocytosis and chemotaxis of macrophages, and reducing cytokine contents. Moreover, hyperglycemia in the body is beneficial to bacterial reproduction, enhances bacterial virulence and accelerates the occurrence of infections.^21^-^23^ Some experts believe that the length of catheter indwelling time and the incidence of infections have a certain relationship, and the infection rate of patients will increase with the increase in catheter indwelling time. With the increase in catheter indwelling time, fibrin sheath is produced on the surface of the lumen, mainly composed of inflammatory factors, fibrin and platelets. Bacteria will migrate and colonize along the tissue around the catheter, and enter the body from the fibrin sheath, increasing the risk of catheter-related infections.^24^-^26^ Therefore, in the nursing of patients with maintenance hemodialysis, we should master the method of sealing catheter, as well as the patients should actively learn the relevant nursing knowledge, avoid scratching the skin at the catheter site, and pay attention to personal hygiene habits. Hypoalbuminemia is associated with immunosuppression, malnutrition and long-term dialysis of patients, and can decrease the bactericidal ability and phagocytosis of macrophages and neutrophils, weaken the activity of NK cells, reduce the activity and quantity of T lymphocytes, and inhibit the differentiation, which will increase the risk of infections.

### Limitations of this study

The number of subjects included in this study was limited, so the conclusions drawn may not be very convincing. In addition, this study was a retrospective descriptive study, with limited clinical data available and limited persuasive conclusions. Further intervention trials are needed in the future to confirm these results.

## CONCLUSION

The main pathogens of patients with ESRF on hemodialysis through permanent catheter CRF are Gram-positive cocci. Hypoalbuminemia, too long catheter indwelling time and diabetes are risk factors for infections. Therefore, in clinical nursing, targeted measures should be taken for patients with high-risk infections, so as to increase the catheterization time and reduce the infection rate.

### Authors’ Contributions:

**Jun Dou** and **Xuebing Wu** designed this study and prepared this manuscript, and are responsible and accountable for the accuracy and integrity of the work.

**Hua Ao** and **Ming Li** collected and analyzed clinical data.

**Qiuling Zhang** significantly revised this manuscript.
